# Dpp-Expressing and Non-Expressing Cells: Two Different Populations of Growing Cells in *Drosophila*

**DOI:** 10.1371/journal.pone.0121457

**Published:** 2015-03-23

**Authors:** Carolina Arias, Gimena Fussero, Marcelo Zacharonok, Ana Macías

**Affiliations:** 1 Departamento de Fisiología, Cátedra de “Genética” Facultad de Ciencias Exactas Físicas y Naturales de la Universidad Nacional de Córdoba, Córdoba, Argentina; University of Bern, SWITZERLAND

## Abstract

There are different models that explain growth during development. One model is based on insect and amphibian regeneration studies. This model proposes that growth is directed by pattern, and growth takes place by intercalation at a growth discontinuity; therefore, proliferation should surround the discontinuity. Currently, this model, apart from regenerative studies on mostly adult patterning, has not found supporting evidence in *Drosophila* that shows proliferation surrounding a discontinuity. Despite this lack of evidence, the importance of discontinuities has been shown in different experiments, even under wt conditions, more specifically in the formation of the leg joints because of the occurrence of cell death at their boundaries. Here, we show the existence of a sharp discontinuity in Decapentaplegic (Dpp) in the genital discs at the third larvae stage (L3), which determines the upregulation in the Jun-NH2-Terminal-Kinase (JNK) pathway, *reaper* (*rpr*), *head involution defective* (*hid*) and active caspases from its boundaries. The proliferation and cell death surrounding the discontinuity suggest that growth can proceed by intercalation and competitive death takes place in this area. Finally, we show that the Rpr, Grim and Hid (RGH) products are a few of the factors that define the growth discontinuity because they are negative regulators of growth, a new function that is unique from their known functions in apoptosis.

## Introduction

French *et al*. and Hayne and Bryant (1976) [1, 2] proposed that pattern directs growth and that growth is derived from the confrontation of different growth positional values through proliferation. Proliferation intercalates the intermediate values. Aside from the relationship between pattern and growth, in this model, proliferation is induced at a discontinuity in growth conditions. Their proposal was based on their studies on insect and amphibian regeneration. For example, when a cockroach leg is cut proximally and grafted with a distal portion of the leg, the entire leg is regenerated by intercalary growth. Since growth and pattern for those days (and now [3]) in *Drosophila* was seen to be due to a gradient, Hayne and Bryant said they could agree if each cell defines its position by two parameters. Considering the two parameters, Müller *et al*. (2003) [4] showed that the Dpp gradient is transformed into an inverse gradient of Brinker (Brk). The Dpp pathway represses the expression of *brk*; in turn, Brk, which is a transcriptional repressor factor, represses the Dpp pathway [5]. Based on these studies [4, 5], Martín *et al*. (2004) [6] showed that Brk is a growth repressor and proposed that growth is the result of the cellular amounts of Dpp and Brk. Although most of the experiments by French *et al*. and Hayne and Bryant (1976) [1, 2] were conducted in the wing disc; at L3, there is no correspondence among the proliferation and the Dpp discontinuity in this disc [5]. Adachi-Yamada and O’Connor (2002) and Moreno *et al*. (2002) [7, 8] contributed other new insights to our knowledge of discontinuities, which is that cell death occurs at their boundaries. In *Drosophila*, the only wt example of the occurrence of death at a Dpp discontinuity is during the morphogenesis of the leg joints [9], and currently, there is no evidence that proliferation occurs at a Dpp discontinuity, which would support the intercalary growth model [1,2].

The genital disc is an appropriate structure for demonstrating whether discontinuities are important for promoting growth. A time point at which a discontinuity in Dpp is expected is at the beginning of its expression because as development proceeds a discontinuity should be diluted by proliferation and should eventually disappear. Notably, the genital disc stops growing after the embryonic stage and resumes growth during the second larval stage (L2) following the onset of Dpp activation [10]. The transition from L2 to late L3 (the stage at which the discs are analyzed) occurs over 48 h, which is a small amount of time compared with the 96-h period for wing disc growth following Dpp activation.

To analyze discontinuities we focused on JNK, the pro-apoptotic genes *reaper* (*rpr*), *grim*, and *head involution defective* (*hid*) (RGH). We analyzed these factors in relation to Dpp expression/activity. JNK is a mediator of *Drosophila* apoptosis [7, 8]; accordingly, it is a transcriptional activator of the pro-apoptotic genes *hid* and *rpr* [11, 12] and may regulate the others such as *grim*, *sickle* and *jafrac2*. The RGH products are positive activators of caspases [13], and they are the effectors of apoptosis [13]. RGH promote the activation of caspases by competing for the binding sites in Drosophila Inhibitor of Apoptosis 1 (DIAP1), which liberates the caspases to be activated and promote death [13]. Rpr and Grim are also inhibitors of translation, and this function is considered part of the apoptosis process [14]. RGH are subject to diverse negative regulatory mechanisms at the transcriptional [15], post-transcriptional [16] and post-translational [15] levels, which reflects the importance of regulating the RGH levels.

Based on our experiments in the genital disc, we identified two distinct populations of growing cells that either express or do not express Dpp. The lack of Dpp expression coincides with a sharp boundary of Dpp activity, high JNK activity and elevated expression levels of *rpr*, *hid* and active caspases. In other words, the JNK\RGH\caspase expression patterns are complementary to Dpp. At the boundary of these populations, we observed cell division and death; moreover boundaries with these characteristics were formed when we manipulated the Dpp expression. All of these data indicate the existence of a discontinuity in the growth conditions among the cells that express or not express Dpp at L3 in the genital discs. The location of proliferation suggests that growth proceeds by intercalation. Finally, we observed that the RGH products affect proliferation by repressing growth, and this is a new function that is independent of apoptosis but is highly important when a cell is targeted for death.

## Materials and Methods

### Genetic strains

Gene expression experiments were performed using the Gal4/UAS method [17]. The drivers used were *Abd-B-*Gal4^LDN^, a *Gal4* insertion into the *Abd-B m* enhancer [18] (referred to as A8), and the promoter of Dpp linked to the Gal4 gene [19]. The following UAS transgenes were used: UAS-EGFP (Bloomington Center) (referred to as GFP) and UAS-Dpp-GFP [20]. The reporter genes used were as follows: *puc-Z*, which is present in the *puc* ^*E69*^ allele [21], the *hid* allele W^05014^ (Bloomington Center), and the 4 kb *rpr-lac*Z construct [22].

### Overexpression conditions and clonal analysis

Clone mutants for *Df(3L)H99* [23] were induced in larvae with the following genotype: *y*, *w*, *hs-flp/+*; *Df(3L)H99* FRT2A/*Ubi*–GFP, FRT2A [24]. The control larvae were *y*, *w*, *hs-flp/+*; FRT2A/*Ubi*–GFP, FRT2A. The crosses were maintained at 25°C, and the clones were induced at L2, heat-shocked at 40°C for 15 minutes and then dissected for histochemical analysis at L3. Statistical analyses were performed using the InfoStat software program [25] and Student’s t-test.

### Antibodies and immunohistochemistry

The following primary antibodies were used: rabbit anti-active human caspase 3 (Cell Signaling Technology), mouse anti-β-Gal (Sigma), rabbit anti-β-Gal (Promega), rabbit anti-human histone 3 (pSer10) (Lifespan Biosciences), anti-bromodeoxyuridine (Santa Cruz Biotechnology), anti-Wingless (a gift from Morata G), anti-Engrailed (a gift from Sánchez-Herrero E), and anti-Brinker (a gift from Martín F). We used fluorescent secondary antibodies from Invitrogen. The TUNEL reaction was performed using an *in situ* apoptosis detection kit from Roche. Nuclei were detected by including propidium iodine (PI) or DAPI in the mounting media (Vectashield PI-Vector Labs). For the detection of nuclei with PI, the dissected/fixed samples were treated with RNAase (30 μg/ml) for 30 minutes at 37°C prior to immunostaining.

### Image collection and processing

Fluorescent images were obtained using Zeiss or Olympus FV1000 confocal microscopes during multi-track sessions and processed using the LSM 5 Image Browser, FV10-ASW4.0 Viewer, MacBiophotonic, ImageJ and Adobe Photoshop CS4 software programs.

### Ethics statement

No specific ethics statements were required for this work. This report does not describe research involving human participants. The present version of this paper has the verbal approval of all the authors, who examined both the text and illustrations in detail. The authors declare that this manuscript has not been submitted simultaneously for publication in any other journal nor have the findings been partially disclosed in any other publication.

## Results

### Discontinuities in Dpp: activated JNK and caspases

**Fig 1:**
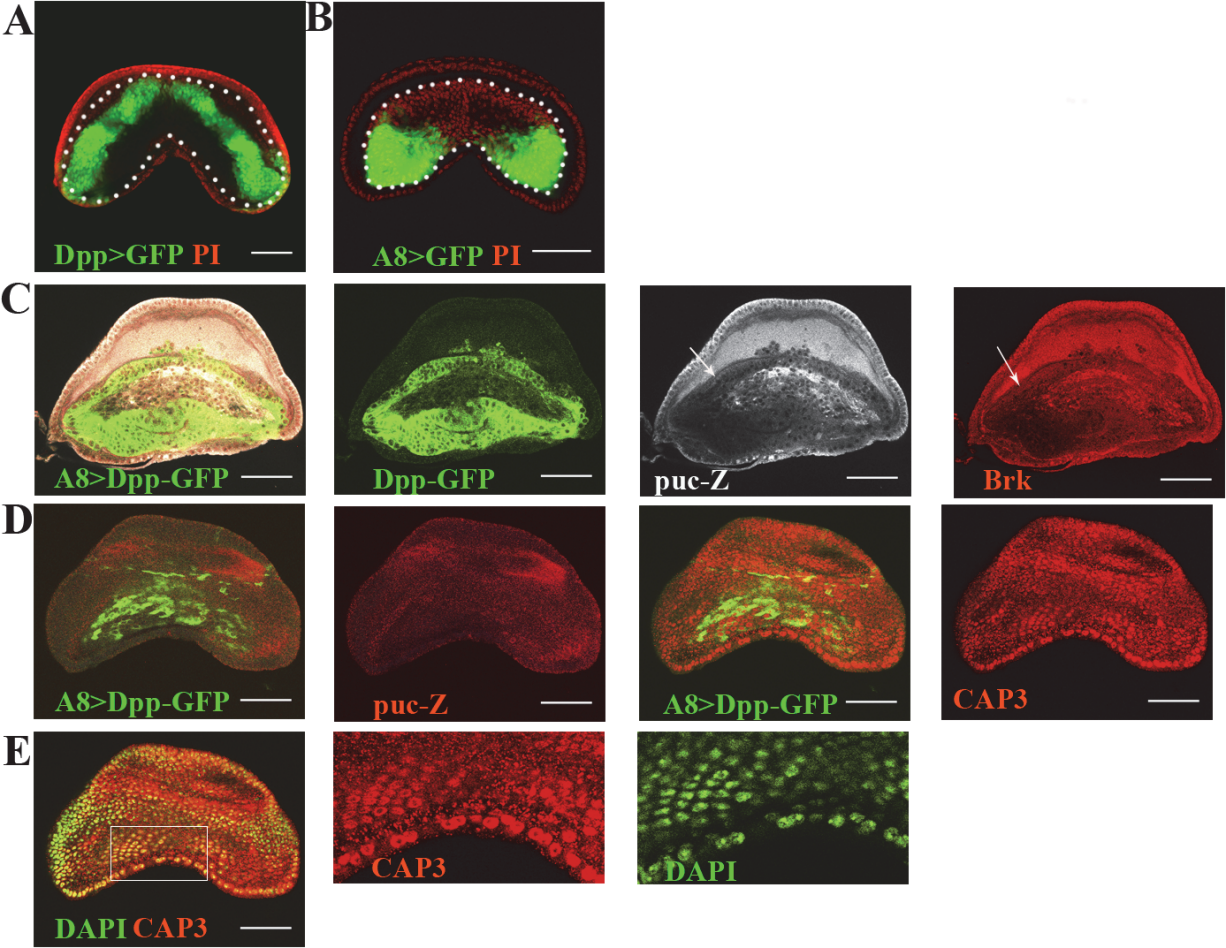
Activation of JNK and caspases by overexpressing Dpp in the female A8 primordium. In all of the figures, the white bars correspond to 50 μm. **A-B)** The expression patterns of GFP driven by Dpp-Gal4 and A8-Gal4. The A8 primordia are indicated with white dots. Note that the expression driven by the A8-Gal4 line is different from the endogenous Dpp expression pattern. **C-E)** Dpp overexpression in the female A8 primordium revealed by GFP-fused to Dpp. **C)** JNK activity revealed by puc-Z borders the Dpp-overexpressing cells. The JNK activity pattern coincides with the expression pattern of Brk. Note that the upregulation of JNK and Brk begin in the Dpp-expressing cells before the border (arrows). **D)** The expression pattern of active caspase 3 (CAP3) overlapped with the JNK activity pattern. **E)** The same disc and section shown in **D)** now showing the nuclei with DAPI and the expression of CAP3, indicating that these cells are apoptotic (shown in the highlighted and augmented area).

To analyze whether discontinuities in the Dpp gradient activate JNK [7] and, in turn, the caspases, we overexpressed Dpp using the Gal4/UAS method [17]. We overexpressed this gene in the female A8 primordia using the A8-specific driver *Abd-B-*Gal4^LDN^ [18] (referred to here as A8), which is expressed in a pattern that is distinct from that of endogenous Dpp (Fig. 1A). Consequently, this overexpression should disrupt and create discontinuities in the endogenous Dpp gradient. As observed in the wing disc [7], the discontinuities generated from this overexpression were accompanied by an upregulation in JNK at the boundary of ectopic Dpp expression (Fig. 1C), confirming that Dpp is a repressor of JNK [26], and JNK appeared as a target of Dpp pathway. Consequently, this boundary is not only a boundary of Dpp expression but is also a boundary of activity. In support, the Dpp target Brinker (Brk), which is negatively regulated by Dpp [5], is also upregulated at this location (Fig. 1C), similar to JNK and indicative of the role of Brk as a transcriptional repressor of the Dpp pathway [5].

Next, we determined whether JNK upregulation also serves to activate the caspases, and indeed, we observed active caspases in a similar pattern to that of active JNK (Fig. 1D). Interestingly, however, active caspases were also detected in the nuclei (Fig. 1E), indicating that these cells were apoptotic and confirming that discontinuities promote death that can be competitive at their boundaries [7, 8, 9].

The patterns in upregulated JNK and caspase activity precisely followed the borders of Dpp overexpression. Therefore, we next investigated the expression patterns of JNK, *rpr*, *hid* and the caspases in relation to Dpp expression under wt conditions.

### The relative expression patterns of Dpp, JNK, *rpr*, *hid* and the caspases

Dpp expression was visualized by the expression of GFP, which was driven by a construct of the Dpp promoter link to the Gal4 gene [19]. JNK activation was analyzed in an indirect manner. We utilized a *lac-Z* insertion into the *puckered* locus (allele *puc* ^*E69*^) called puc-Z to monitor JNK activity [21] because *puc* is a transcriptional target of JNK in *Drosophila* [21]. However, Puc is also a negative regulator of JNK, which raises the question of whether *puc* expression accurately represents JNK activity. Regardless, McEwen and Peifer [27] analyzed *puc* function and concluded that JNK is active in all cells, at least at a basal level. The expression patterns of *rpr* and *hid* were analyzed using *lac-Z* reporter genes fused to their respective promoters. For *rpr*, we used a 4 kb construct that contained the *rpr* promoter and reproduced the endogenous expression pattern [22]. For *hid*, we utilized a *lac-Z* insertion into the *hid* gene, which also causes a mutation. Adult flies carrying any of these constructs alone or in combination under heterozygous conditions were normal, indicating that the constructs do not affect growth. To observe the expression pattern of active caspases, we used the anti-active human caspase 3 (CAP3) antibody, which also cross-reacts with the effector caspase Drice.

**Fig 2:**
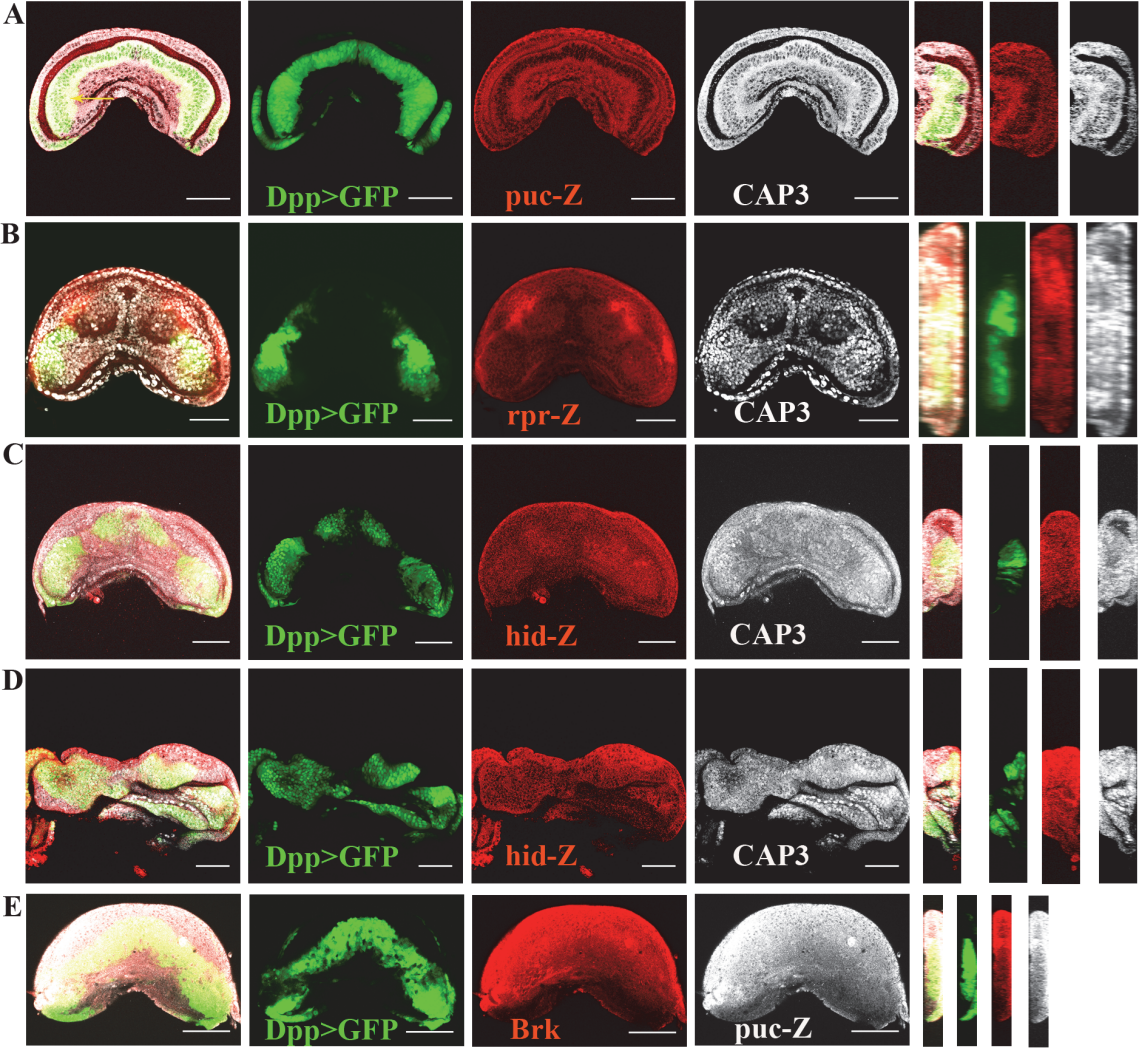
Comparative expression patterns of JNK, *rpr*, *hid* and Brk in relation to Dpp and CAP3 or JNK in the genital disc at L3. Dpp expression was visualized by GFP. The regulatory relationships between the factors are revealed by their expression patterns, which are highlighted in cross sections. All of the discs are females with the exception of **D)**. **A)** Dpp expression is surrounded by high levels of JNK activity, which coincides with the pattern of active caspases. Note that the high activity of JNK begins before the border of Dpp-expressing cells (yellow arrow). **B)** *rpr* and consequently the active caspases are upregulated along the borders of Dpp expression. **C-D)** *hid* and consequently the active caspases are upregulated near the borders of Dpp expression. Note that in the female shown in **C)**, *hid* is more weakly expressed than it is in the male shown in **D)**. **E)** Brk is upregulated from the border of Dpp expression and overlapped with the high levels of JNK activity.

We observed that all of these factors demonstrated similar patterns of expression; specifically, they were expressed at low levels in the center of the Dpp expression domain and were more strongly expressed closer to the Dpp boundary (Fig. 2A-D and S1 Fig. A-B). This endogenous expression pattern is similar to the pattern we observed when we overexpressed Dpp and analyzed the expression of JNK and the caspases (Fig. 1C-E), with the exception that the caspases were mostly cytoplasmic. Importantly, the expression level of *hid* is lower in females than in males (Fig. 2 compare C with D), which is consistent with previous results and suggests a less important role for *hid* in females [12]. Because the expression patterns suggest that the border of Dpp expression constitutes a sharp discontinuity in activity, we evaluated the expression of Brk. Consistent with this speculation, we observed a high level of Brk expression surrounding Dpp expression, which was similar to the other factors analyzed (Fig. 1E and S1 Fig. C).

### Cell death and division at the Dpp discontinuity

**Fig 3:**
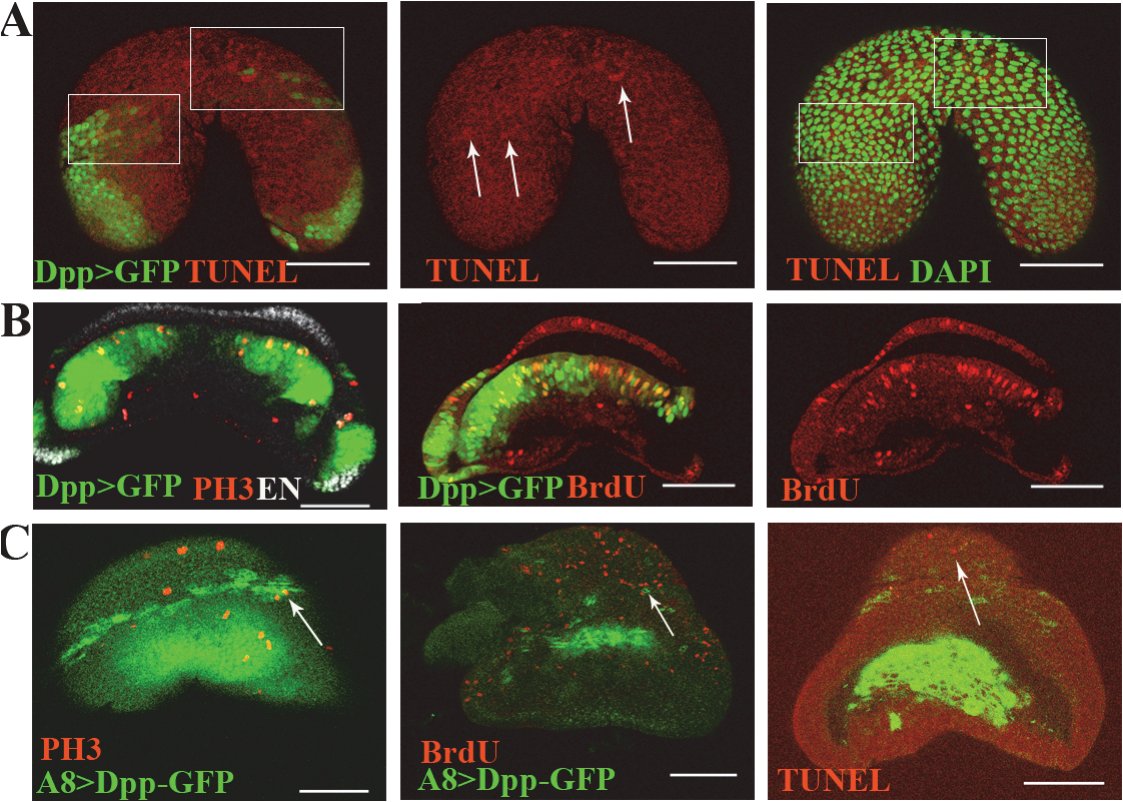
Cell death and division at the border of Dpp expression. Females. **A)** Apoptotic cells were detected adjacent to the Dpp-expressing cells. Note that the apoptotic nuclei are fragmented or exhibit abnormal forms and are highlighted with DAPI. **B)** L3 discs from Dpp>GFP flies showing dividing cells marked with anti-PH3 or BrdU. Note that the divisions occur next to and surrounding the Dpp-expressing cells. **C)** L3 discs from A8> Dpp-GFP flies stained with anti-PH3 or BrdU. Dpp-directed expression induces cell divisions at the borders of overexpression. Note, the individual cells expressing Dpp with an adjacent dividing cell, arrows. **D)** L3 discs from A8> Dpp-GFP flies with TUNEL staining; note, the apoptotic cell adjacent to the Dpp-expressing cell (arrow).

Based on previous results [7, 8, 9], we hypothesized that there would be cell competition at the wt Dpp discontinuity. Therefore, we assayed for apoptosis in this region. We examined 30 discs using the TUNEL assay and observed very little apoptosis (Fig. 3A and S2 Fig. A). One problem with detecting apoptotic cells could be that they are quickly removed or engulfed by their neighbors [28].

We analyzed the occurrence of cell division, which should occur in the area surrounding Dpp expression because discontinuities may induce proliferation [1, 2] and because some proliferation may also be associated with death [29, 30, 31]. Indeed, we found that all of the cell divisions occurred in the area surrounding the Dpp expression domain (Fig. 3B and S2 Fig. B). In agreement with this finding, when we manipulated Dpp expression to create a great discontinuity, the cell divisions occurred in the area surrounding it. Moreover, isolated Dpp-expressing cells were accompanied by adjacent dividing cells (Fig. 3C). In males, this procedure yielded even more striking results; the isolated Dpp-expressing cells outside of the A8 domain were often accompanied by dividing cells (S2 Fig. C).

### Cell death at the Dpp discontinuity and the proliferative function of the RGH products

It is known that homozygous and even hemizygous DfH99 cells can not always execute cell death [32]. Therefore, these types of cells (“undeath cells”), which are targeted to die but are unable to complete the process, keep secreting the growth molecules Dpp and Wingless (Wg) [29, 30, 31] and because of this mechanism, they are easily detected. Although we showed that the pro-apoptotic genes *rpr* and *hid* are expressed in the area surrounding the Dpp discontinuity, we scarcely observed death in this area. Therefore, we decided to induce genetic mosaics using the H99 deficiency (DfH99), which removes all of the RGH genes. In this experiment, three populations of cells with different levels of RGH were grown together, and if clones or twins at the Dpp discontinuity were targeted for death, only the twins should die and the clones should stay as “undeath cell”.

**Fig 4:**
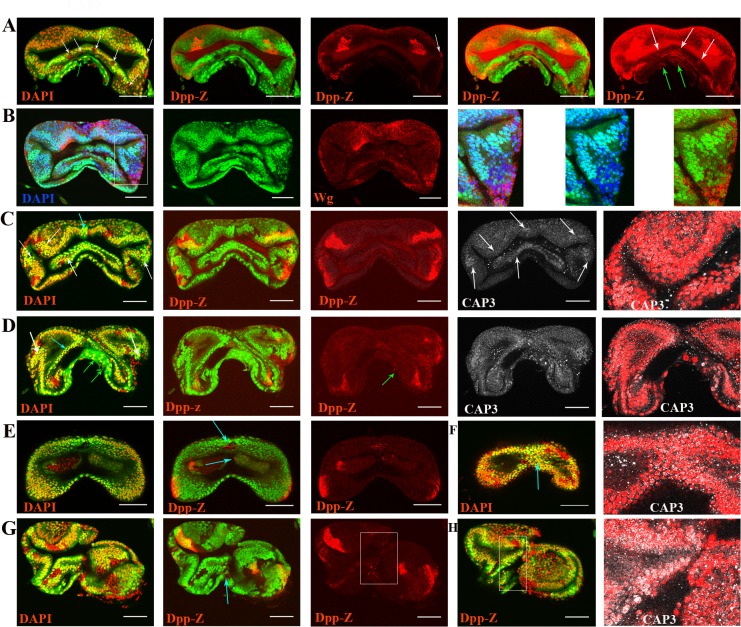
Death at Dpp discontinuity, at the midline and at the larvae stalk cells. All the samples were males with the exception of **E)**. **A)** Undeath cells at the Dpp discontinuity (white arrows) and at the larvae stalk (green arrow). Note that the clones, “undeath cells,” were detected by their expression of Dpp. **B)** Clones, “undeath cells,” at the Dpp discontinuity were revealed by their expression of Wg. **C**-**D)** Death of the twin cells at the Dpp discontinuity (white arrows), at the midline (light blue arrow) and at the larvae stalk cells (green arrows). Note that death is marked by the CAP3-positive nuclei. **E-H)** Death at the midline (light blue arrows and highlighted areas) clones: in the form of “undeath cells” expressing Dpp; twins: showing CAP3+ nuclei **E, G)** “undeath cells” **F, H)** twins death.

Consequently, we investigated cell death in relation to the Dpp discontinuity. Indeed, we found clone undeath and twin cell death at the Dpp discontinuity (Fig. 4A white arrows, B highlighted area, C-D white arrows), confirming the occurrence of cell death at the Dpp discontinuity. Additionally, we also observed death in the larvae cells (Fig. 4A-D, I green arrows) and at the midline (Fig. 4C-G light blue arrows and highlighted area in G-H). The death that occurred at the midline appeared to be necessary to establish symmetry, because the induction of DfH99 clones and the prevention of death by other means (i.e., downregulation of JNK and caspases, data not shown) produced asymmetric growth (Fig. 4C, D, F, G, H).

**Fig 5:**
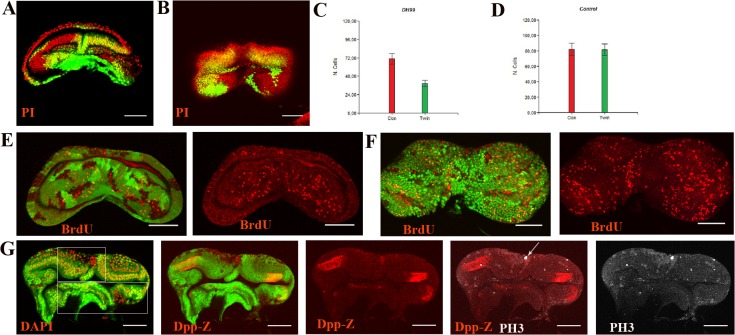
The RGH products repress proliferation. **A)** Female and **B)** male. DfH99 clones are marked by PI and a lack of GFP and twins are marked by two doses of GFP. The growth of the clones exceeded that of the twins and the cells with the genetic background. **C)** The DfH99 clones grew on average 50% larger than their respective twins (P<0.05). **D)** The control clones and their respective twins grew similarly (P>0.05) **E)** Female. **F)** Male. Note the correlation between the cell divisions with the clones and the cells with the genetic background, but not with the twins, dividing twin cells occur only at the borders. **G)** Male. Note the dividing clone cell next to an “undead cell” expressing Dpp (arrow). Also note the PH3 expression revealing not only the whole chromosomes, the huge positive spots, but also previous stages of Histone 3 phosphorylation, the fine positive spots. The fine positive spots surround the Dpp expression and are more abundant in the clones than in the twins.

Surprisingly, the DfH99 clones grew a lot (45 pairs) (Fig. 5A-B) on average; they were 50% larger than the twins, and occasionally, twins were not present, whereas in the controls, both cell types were similar in size, and the twins were always present (Fig. 5C-D). This finding suggests that the RGH products act over growth by repressing proliferation and being factors of competition when their levels differ among neighboring cells. Consistent with this speculation, cell death was observed in the twins (Fig. 4C, D, F, and H). However, the twins appeared to be subject to double cell competition with the clones in any part of the disc and with cells of the genetic background at the Dpp discontinuity, which explains the common absence of the twin.

As a follow-up to these experiments, we examined the locations of the dividing cells and expected that the locations would not correspond with the twins. Indeed, this was the case; proliferation corresponded with the clones and the cells of the genetic background but not with the twins (Fig. 5E-F). Furthermore, if proliferation was present in the twins it was at their borders, suggesting that proliferation was associated with death [29, 30, 31]. In agreement with the notion that proliferation is associated with death, we found that proliferation corresponded with the “undeath cells” (Fig. 5G arrow) but more frequently perfectly correlated with the PH3-positive points, which are indicative of the initial stages of phosphorylation and the commitment to divide (Fig. 5G). Interestingly, these PH3-positive points surrounded the Dpp discontinuity and were more concentrated in the clones than in the twins (Fig. 5G compare in the highlighted areas), which is in agreement with the notion that discontinuities induce proliferation and the RGH products repress proliferation.

## Discussion

Our results showed that in the genital discs, a discontinuity in growth conditions exists that coincides with the border of Dpp expression. The discontinuity was characterized in different ways. Additionally, we provide evidence that the RGH products negatively affect proliferation, thus limiting growth, through a mechanism that is not necessary linked to apoptosis.

### Discontinuity characterization

The wt border of Dpp expression coincides with a sharp boundary of activity, which is shown by the expression of Brk. This finding confirms that there is a discontinuity in the growth conditions because Dpp and Brk repress each other and are positive and negative regulators of growth, respectively [5, 33, 6]. Additionally, we also characterized the discontinuity by showing the upregulation of JNK and its targets *rpr* and *hid*. In accordance with the expression of *rpr* and *hid* we detected death there (analyzed below), but we also showed these products are influencing the discontinuity because of their effect over proliferation. In this case, cells expressing less RGH, such as clones and cells with the genetic background, proliferate more than the cells with higher levels of RGH, such as the twins. The biochemical studies showed that *grim* and *rpr*, but not *hid*, are inhibitors of translation [14], an activity that is directly correlated with growth. Although *hid* was shown to not be involved, it has been reported that *hid* promotes the increase in the *rpr* level [15]. One possibility is that the effect of Brk over growth[6] may be the result of the RGH products. Brk is a repressor of Dpp, which is a repressor of JNK; therefore, the activity of Brk favors the activation of JNK. Additionally, Brk is also a repressor of *bantman (ban)*, the miRNA that acts directly on the *hid* mRNA and indirectly on *rpr* [16]. *ban* promotes proliferation and inhibits apoptosis [16]; functions that perfectly correspond with its effects on *hid* and *rpr*. In summary, our results on the RGH products are consistent with previous data [4, 5, 6, 16] and support this new role of RGH, which reinforces the existence of the discontinuity because of the sharp differences in the RGH products observed. Despite the RGH participate in the two fundamental processes of growth, proliferation and death, they do not appear to be involved in patterning because the DfH99 clones neither produce duplications nor overgrowth, like Dpp certainly does [33, 34].

The occurrence of death resulting from competition at discontinuities has been well established [7, 8, 9]. In agreement with this mechanism, we showed death that can be competitive using clone “undeath cells” and twin death cells at the Dpp discontinuity site. The “undeath cells” are not targeted to die because of competition with the twins; since in that case, death is directed to the twins. The competition is established with cells of the genetic background that differs probably in the amount of Dpp. The occurrence of this death correlates with proliferation [29, 30, 31]. Thus, part of the proliferation that occurs in the area surrounding the Dpp discontinuity corresponded with proliferation that is associated to death.

With our strategy of using DfH99-induced clones to detect death, we found death in other areas than at the discontinuity. We specifically refer to the death occurring at the midline. The physical separation of the two halves appears to be necessary for symmetric growth. Supporting this proposal, the DfH99 clones produce asymmetric growth. It has been demonstrated that the mechanism controlling organ size growth does not count cells it counts distances [35]. Thus, because the growing area is at the discontinuity, the distance measured is there, and may consist in the dilution of the discontinuity, generating in between, all of the intermediate growth positional values.

Additionally, defining the discontinuity, there is the surrounding proliferation. This proliferation is induced by the discontinuity because it appeared at the same location when we changed the Dpp expression to generate a discontinuity. This proliferation, does not completely correspond with the proliferation associated with death [29, 30, 31] because in the DfH99 clones, in which the death-targeted cells are unfailingly marked, we observed much more proliferation not related to death. It has been proposed that proliferation at the same time dilutes the differences in growth promotes growth [1, 2]. The existence of a discontinuity surrounded by proliferation supports this proposed mode of growth. However, the model proposed, it is the pattern whom directs growth, and we remain to be demonstrate this, but since in Drosophila the pattern and growth are directed by the same molecules, and one of this is Dpp [34], it is highly probable that this relation exists.

Finally, we want to discuss aspects of JNK regulation and activity. We previously mentioned that Dpp acts as a repressor of JNK; however, the up-regulation of JNK begins in cells that express Dpp at the border, indicating that there is a JNK activator that counteracts the effect of Dpp in these cells. This observation is supported by the overexpression of Dpp shown in this work and the lack or constitutive activity of the Dpp pathway [7] that upregulates JNK in the same nonautonomous way. This factor marks the limits of the discontinuity; it may depend on JNK and has a short range of activity. Although JNK appeared highly repressed in the Dpp domain, we observed that the clones and twins grew there, maintaining their growth differences, demonstrating the function of JNK\RGH. In conclusion, there are JNK activity all over the discs at different strength.

## Supporting Information

S1 Fig Comparative expression patterns of JNK, *rpr* and Brk in relation to Dpp and CAP3 or JNK in the genital disc at L3 in males. **A)** High levels of JNK activity are detected at the border of Dpp expression; the activity of JNK is reflected in the activation of caspases. **B)** *rpr* expression surrounded the Dpp expression and coincided with high levels of active caspases. **C)** Brk is upregulated at the border of Dpp expression and overlapped with the high levels of JNK activity.
(TIF)

S2 Fig Cell death and division border the Dpp expression. Males. **A)** Apoptotic cells are detected adjacent to the Dpp-expressing cells. The apoptotic nuclei were fragmented or exhibited abnormal forms. **B)** L3 discs from Dpp>GFP flies showing the dividing cells marked with anti-PH3 or BrdU. Note that the divisions occur next to and surrounding the Dpp-expressing cells. **C)** L3 disc of A8>GFP flies showing the GFP expression outside of the A8 primordium, arrows. **D)** L3 discs from A8> Dpp-GFP flies with anti-PH3, the individual A8 cells expressing Dpp outside of the A8 primordium with adjacent dividing cells.
(TIF)
