# Supplementary figures and images for: Dpp-Expressing and Non-Expressing Cells: Two Different Populations of Growing Cells in Drosophila
Source: PLoS One. 2015 Mar 23;10(3):e0121457. doi: 10.1371/journal.pone.0121457 (PMC4370563; doi:10.1371/journal.pone.0121457)

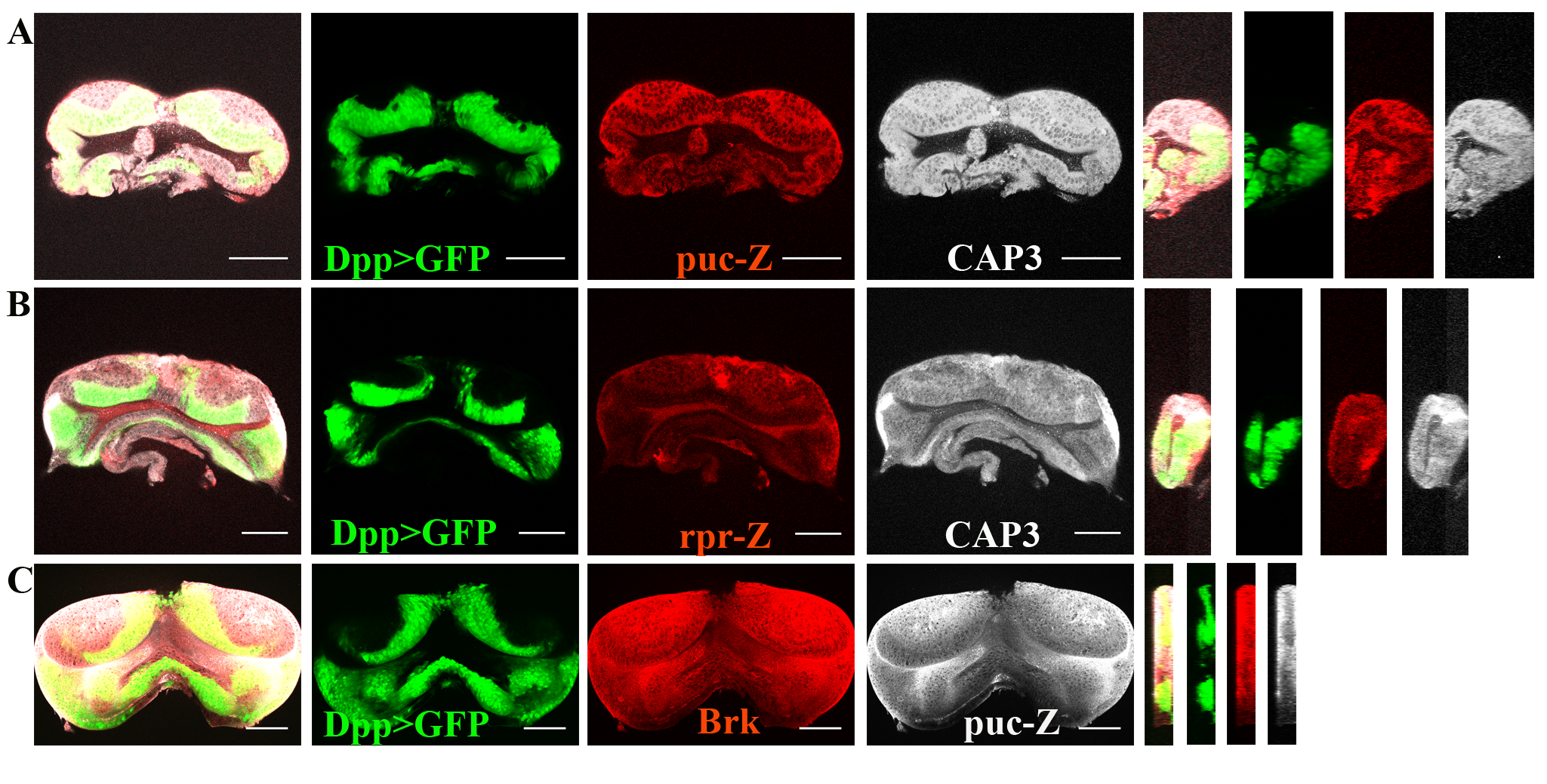

Supplement: S1 Fig — A) High levels of JNK activity are detected at the border of Dpp expression; the activity of JNK is reflected in the activation of caspases. B) rpr expression surrounded the Dpp expression and coincided with high levels of active caspases. C) Brk is upregulated at the border of Dpp expression and overlapped with the high levels of JNK activity. (TIF) [file pone.0121457.s001.tif]

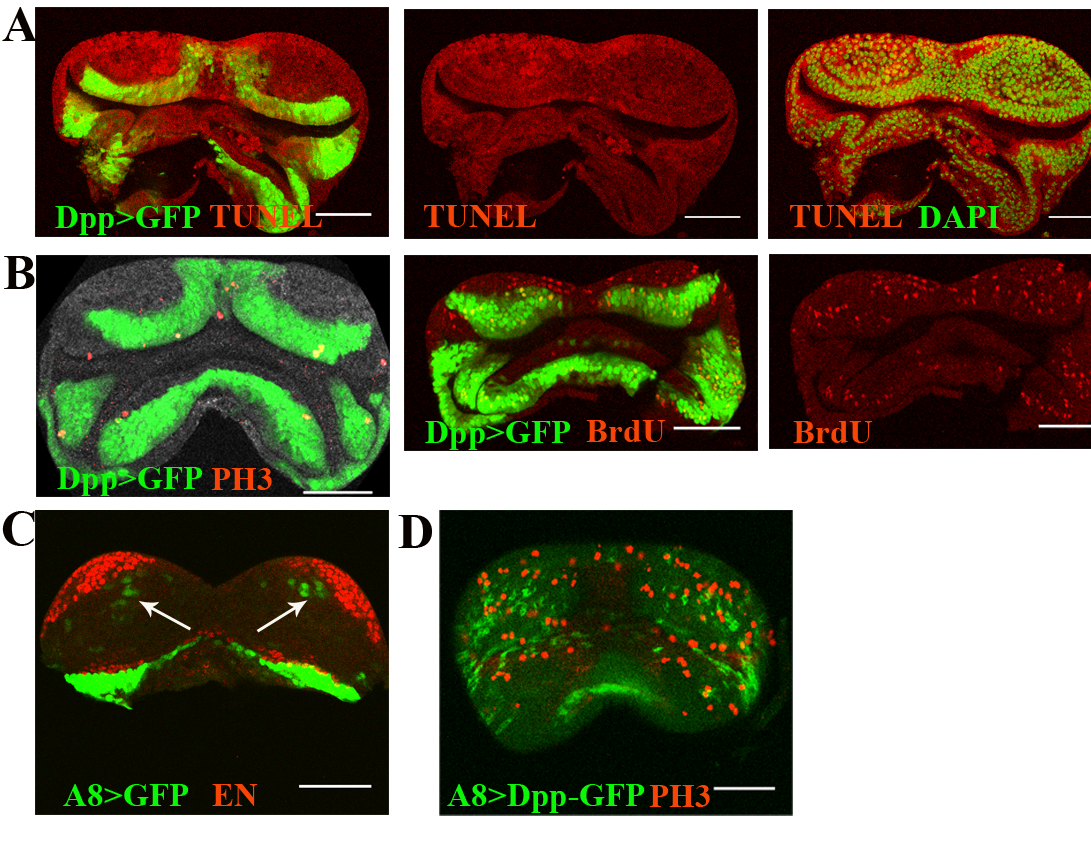

Supplement: S2 Fig — Males. A) Apoptotic cells are detected adjacent to the Dpp-expressing cells. The apoptotic nuclei were fragmented or exhibited abnormal forms. B) L3 discs from Dpp>GFP flies showing the dividing cells marked with anti-PH3 or BrdU. Note that the divisions occur next to and surrounding the Dpp-expressing cells. C) L3 disc of A8>GFP flies showing the GFP expression outside of the A8 primordium, arrows. D) L3 discs from A8> Dpp-GFP flies with anti-PH3, the individual A8 cells expressing Dpp outside of the A8 primordium with adjacent dividing cells. (TIF) [file pone.0121457.s002.tif]
